# Bioinformatic Analysis Reveals Phosphodiesterase 4D-Interacting Protein as a Key Frontal Cortex Dementia Switch Gene

**DOI:** 10.3390/ijms21113787

**Published:** 2020-05-27

**Authors:** Judith A. Potashkin, Virginie Bottero, Jose A. Santiago, James P. Quinn

**Affiliations:** 1 Center for Neurodegenerative Diseases and Therapeutics, Cellular and Molecular Pharmacology Department, The Chicago Medical School, Rosalind Franklin University of Medicine and Science, North Chicago, IL 60064, USA; Virginie.Bottero@rosalindfranklin.edu; 2 NeuroHub Analytics, LLC, Chicago, IL 60605, USA; jose.santiago.ecm@gmail.com; 3 Q Regulating Systems, LLC, Gurnee, IL 60031, USA; jim.quinn@qregulatingsystems.com

**Keywords:** dementia, Alzheimer’s disease, vascular dementia, frontotemporal dementia, switch genes, phosphodiesterase 4D-interacting protein, myomegalin, cardiomyopathy-associated protein 2, valproic acid, inflammation, PI3K-AKT pathway, ubiquitin mediated protein, gene expression, bioinformatic analysis, personalized medicine

## Abstract

The mechanisms that initiate dementia are poorly understood and there are currently no treatments that can slow their progression. The identification of key genes and molecular pathways that may trigger dementia should help reveal potential therapeutic reagents. In this study, SWItch Miner software was used to identify phosphodiesterase 4D-interacting protein as a key factor that may lead to the development of Alzheimer’s disease, vascular dementia, and frontotemporal dementia. Inflammation, PI3K-AKT, and ubiquitin-mediated proteolysis were identified as the main pathways that are dysregulated in these dementias. All of these dementias are regulated by 12 shared transcription factors. Protein–chemical interaction network analysis of dementia switch genes revealed that valproic acid may be neuroprotective for these dementias. Collectively, we identified shared and unique dysregulated gene expression, pathways and regulatory factors among dementias. New key mechanisms that lead to the development of dementia were revealed and it is expected that these data will advance personalized medicine for patients.

## 1. Introduction

Dementia is a syndrome that often manifests itself as a reduction in cognitive function, especially with regards to memory and thinking [1]. Emotional control, social behavioral changes, and motivation also tend to decline in dementia patients [1]. Dementia is often a major cause of chronic disability and dependency among the elderly. According to the World Health Organization, approximately 50 million people have dementia and 20% of those are newly diagnosed patients [1]. Dementia is associated with a wide spectrum of neurodegenerative diseases, including Alzheimer’s disease (AD), vascular dementia (VaD), and frontotemporal dementia (FTD). The different dementias share several genetic, neurochemical, and morphological factors, making their distinction very challenging [2,3]. Compounding this problem is the fact that mixed forms of dementia are often present in a patient [2]. There are currently no known means of preventing dementia and no treatments that slow progression or cure the disorder [1].

AD is the most common cause of dementia and is characterized by the progressive accumulation of neurotoxic amyloid beta plaques and neurofibrillary tangles [4]. Furthermore, AD is the most prevalent neurodegenerative disease worldwide [1]. The progression of AD pathogenesis has been described in three major clinical categories: preclinical AD, mild cognitive impairment, and AD dementia. The most common clinical symptoms observed in AD patients include cognitive impairment and the deterioration of episodic memory and executive functions [1]. While several biomarkers have shown promise in distinguishing these different stages, other major forms of dementia can mimic AD symptoms at early stages, adding further complexity to the diagnosis [5]. 

For instance, VaD is a common dementia form whose pathological and clinical features significantly overlap with those present in AD. VaD is considered the second most common cause of dementia after AD causing approximately 15% of the cases [6]. VaD is characterized by the rapid and progressive cognitive impairment after a cerebrovascular lesion. Notably, cerebrovascular disease is also common among AD patients and approximately 80% of confirmed AD cases that are based on brain autopsies present with brain vascular pathology [7]. In addition to cerebrovascular disease, other factors, including increasing age, diabetes, female sex, and degree of education, are shared between VaD and AD, further challenging the clinical definition and diagnosis of dementia patients. 

Similarly, FTD is a progressive neurodegenerative disease associated with dementia. FTD is characterized by the neuronal death in the brain frontal or temporal lobes, leading to behavioral changes, decline in cognitive and executive functions, and sometimes, the deterioration of language [8]. FTD patients may present a diverse range of behavioral and psychological symptoms including apathy, changes in social behavior, lack of empathy, and cognitive impairment [8]. Behavioral changes are often the first noticeable symptoms in behavior variant frontotemporal dementia (bvFTD), the most common form of FTD [8]. In contrast, behavioral changes tend to occur in the late stages of AD. Clinically, both FTD and AD present with similar symptoms. For example, FTD and AD patients typically present some language problems such as difficulty with producing or comprehending language and aphasia [9]. Other clinical symptoms, however, are unique to each dementia. While FTD patients perform well in visuospatial tests, AD patients present some visuospatial deficits, including problems with drawing, constructions, and orientation early in the development of the disease [9]. In addition, hallucinations and delusions, relatively uncommon in FTD, become relatively common with AD progression.

Network-based approaches have been useful for understanding dysregulated pathways and key genes associated with dementia. Transcriptomic network analysis has revealed altered pathways and important transcription factors in AD [10,11,12,13], VaD [12,14], and FTD [12,15,16]. Such network analyses have been useful for understanding shared and unique pathways across dementia spectrum disorders [12]. Recently, SWItch Miner (SWIM) software, which uses a novel approach to network analysis, was developed for identifying key genes associated with drastic changes in gene expression patterns, for example, the transition from healthy to disease state [17,18]. The SWIM algorithm develops a gene co-expression network from expression data. Whereas many algorithms look for a positive correlation in gene expression, SWIM goes a step further and finds genes that also show a strong negative correlation with their nearest neighbors in the network. Genes are sorted into party hubs (high positive correlation), date hubs (moderate positive correlation), and fight club hubs (negative correlation, the switch genes). Fight club hubs represent a transitional function from one cell state to another. SWIM analysis has been useful for identifying switch genes involved in the differentiation of glioblastomas [18]. We recently applied SWIM analysis to gene expression data in order to identify genes whose expression is associated with drastic changes in the hippocampus and posterior cingulate cortex of the brains of AD patients [11]. 

In this study, we used the SWIM algorithm to identify genes in the frontal cortex associated with the transition from a healthy aging brain to a state of dementia. We further compared the dysregulated pathways and transcriptional factors involved in AD, VaD, and FTD. The results from this study indicate that phosphodiesterase 4D-interacting protein is a key factor involved in the development of dementia and suggest possible therapies that may be useful in preventing or delaying dementia.

## 2. Results

### 2.1. Database Mining for Brain Transcriptomic Studies 

**Figure 1:**
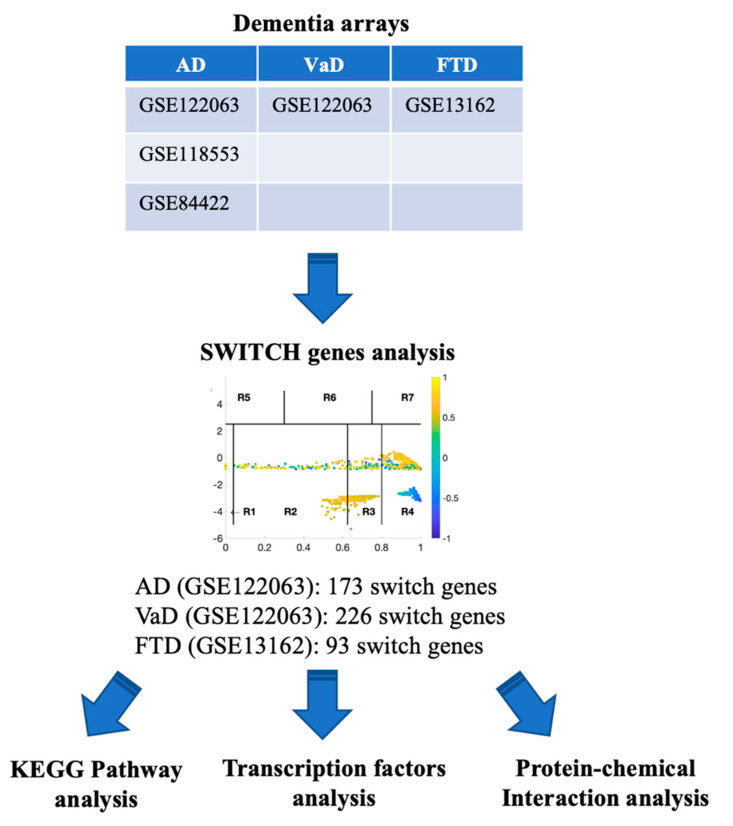
Flowchart of the study. Array Express, NCBI GEO and BSCE databases were searched for frontal cortex transcriptomic studies with data from AD, VaD and FTD patients. Microarrays were analyzed using the SWIM algorithm to identify switch genes. Pathway and transcription factor analyses of switch genes were performed using NetworkAnalyst.

A flow chart of the approach we used in this study to identify and analyze key switch genes and pathways is presented in Figure 1. We searched the Array Express, NCBI GEO, and Base Space Correlation Engine databases to identify studies that contained expression data from postmortem brain tissue from AD, VaD, and FTD patients and age-matched controls. We focused our analysis on the frontal cortex brain region since this area is frequently affected in dementia. Four microarrays contained transcriptomic data from frontal cortex (GSE122063, GSE118553, GSE84422, GSE13162). Details of the datasets analyzed in this study are presented in Table 1. 

### 2.2. Identification of Switch Genes in AD, VaD, and FTD

The SWIM algorithm was used to analyze the data from all the microarrays listed in Table 1 in order to identify key genes that may be associated with the transition from a healthy aging brain to a state of dementia, as described previously [11,17,18]. Only the datasets GSE13162 and GSE122063 were robust enough to identify switch genes after adjusting for the required fold change and *p*-value and, therefore, these data sets were analyzed further (See Methods). 

**Figure 2:**
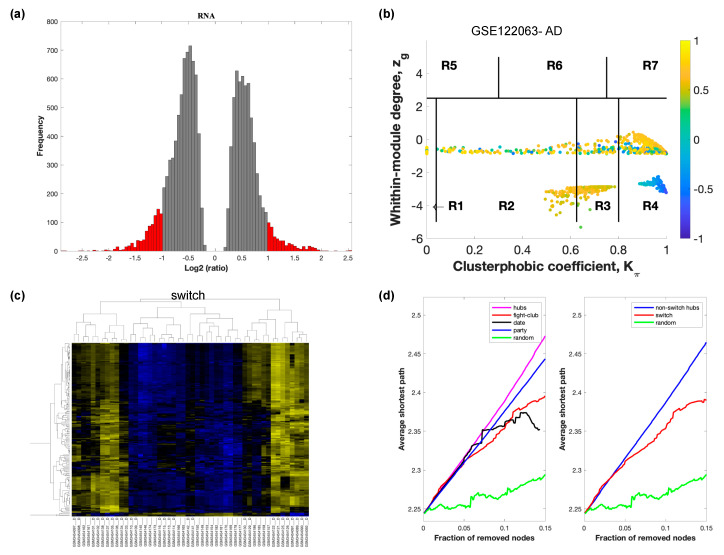
SWIM analysis of the frontal cortex of AD subjects in GSE122063. (**a**) Distribution of log2 fold change values where the red bars are selected for further analysis. (**b**) Heat Cartography Map with nodes colored by their average Pearson Correlation Coefficient. Region R4 represents the switch genes. (**c**) Dendrogram and heat map for switch genes. The suffix _D indicates the sample came from the diseased cohort. (**d**) Robustness of the correlation network.

**Figure 3:**
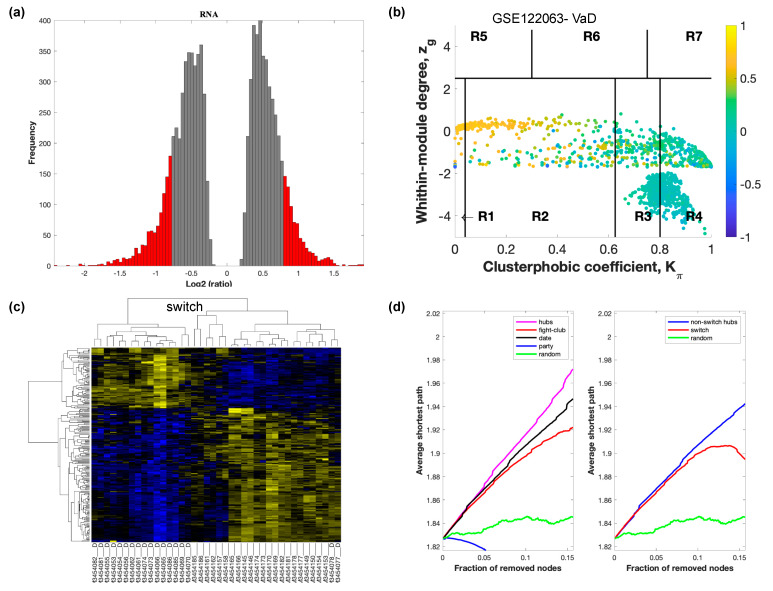
SWIM analysis of the frontal cortex of VaD subjects in GSE122063. (**a**) Distribution of log2 fold change values where the red bars are selected for further analysis. (**b**) Heat Cartography Map with nodes colored by their average Pearson Correlation Coefficient. Region R4 represents the switch genes. (**c**) Dendrogram and heat map for switch genes. The suffix _D indicates the sample came from the diseased cohort. (**d**) Robustness of the correlation network.

**Figure 4:**
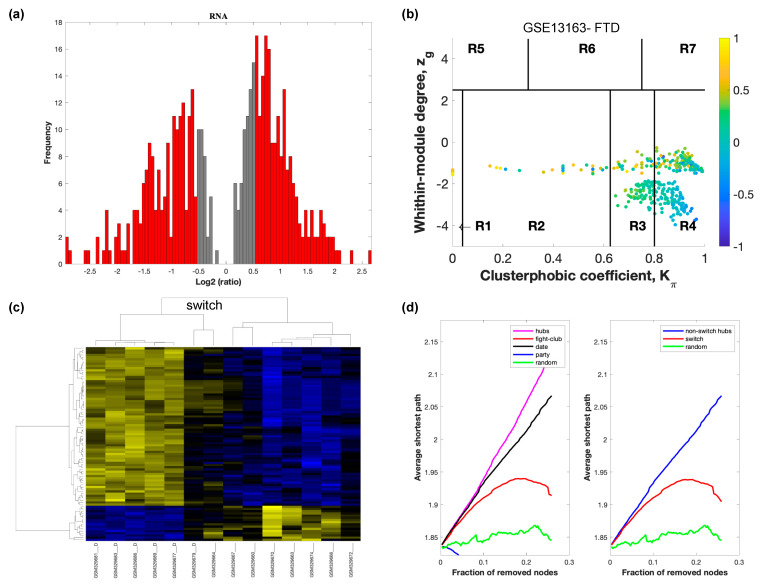
SWIM analysis of the frontal cortex of FTD subjects in GSE13162. (**a**) Distribution of log2 fold change values where the red bars are selected for further analysis. (**b**) Heat Cartography Map with nodes colored by their average Pearson Correlation Coefficient. Region R4 represents the switch genes. (**c**) Dendrogram and heat map for switch genes. The suffix _D indicates the sample came from the diseased cohort. (**d**) Robustness of the correlation network.

The selected raw gene expression datasets from frontal cortex brain tissue from demented individuals were imported into SWIM. We first analyzed the dataset GSE122063 containing samples from AD subjects and age matched healthy controls. The results are shown in Figure 2. Figure 3 depicts VaD patients from dataset GSE122063 and Figure 4 shows the results for FTD patients from dataset GSE13162.

In part (a) of each of these figures, the group of genes that were eliminated and retained according to the selected threshold are depicted as grey and red bars, respectively. The x-axis represents the fold-change value (log2 of the fold-change), that is the ratio of the average expression data in AD patients compared to the average expression data in normal controls, computed for protein-coding and non-coding RNAs. The y-axis represents the frequency of the obtained fold-change values. 

Part (b) of each of these figures, shows the identified correlation communities based on the average Pearson correlation coefficient. The nodes with a negative correlation value with their interaction partner, known as fight-club hubs, are depicted in R4 in blue. The plane is identified by two parameters: *Zg* (within-module degree) and *Kπ* (clusterphobic coefficient) and it is divided into seven regions each defining a specific node role (R1-R7). High *Zg* values correspond to nodes that are hubs within their module (local hubs), whereas low *Zg* values correspond to nodes with few connections within their module (non-hubs within their communities, but they could be hubs in the network). Each node is colored according to its average Pearson correlation coefficient (APCC) value. Yellow nodes are party and date hubs, which are positively correlated in expression with their interaction partners. Blue nodes are the fight club hubs, which have an average negative correlation in expression with their interaction partners. Blue nodes falling in the region R4 are the switch genes, which are characterized by low *Zg* and by high *Kπ* values and are connected mainly outside their module. 

Part (c) of each of these figures shows a dendrogram and heat map of the expression of the switch genes. The expression profiles of switch genes (including protein-coding and non-coding RNAs) are clustered according to rows (switch genes) and columns (samples) of the switch genes expression data (biclustering). The colors represent different expression levels that increase from blue to yellow. In general, the switch genes are upregulated in AD and FTD, whereas they are downregulated in VaD. The results show that the expression of the switch genes in FTD and VaD, cluster the majority of the diseased cohorts together and are distinguishable from the healthy samples. Interestingly, the expression of the AD switch genes does not clearly separate the disease cohort from controls in sample where the fold change is closer to two. However, there is a clear separation in samples in which the fold change is greater than two.

Part (d) shows fight-club hubs differ from date and party hubs and switch genes are significantly different than random, thereby confirming the robustness of the analysis. The x-axis represents the cumulative fraction of removed nodes, while the y-axis represents the average shortest path. The shortest path between two nodes is the minimum number of consecutive edges connecting them. Each curve corresponds to the variation of the average shortest path of the correlation network as a function of the removal of nodes specified by the colors of each curve. The results show that, for AD, VaD, and FTD, the fight club hubs are readily discernable from date and party hubs.

SWIM analysis identified 173 switch genes associated with drastic changes in gene expression in the frontal cortex of AD patients (Supplementary Table S1). The same analysis was performed using the datasets from VaD and FTD patients (Figure 3 and Figure 4, Supplementary Tables S2 and S3). SWIM analysis of gene expression datasets from VaD and FTD patients identified 226 and 93 switch genes, respectively, potentially involved in the transition from healthy aging brain to a dementia phenotype (Supplementary Table S4). These results show that each of these dementias have very few shared changes in gene expression that trigger the dementia. 

**Figure 5:**
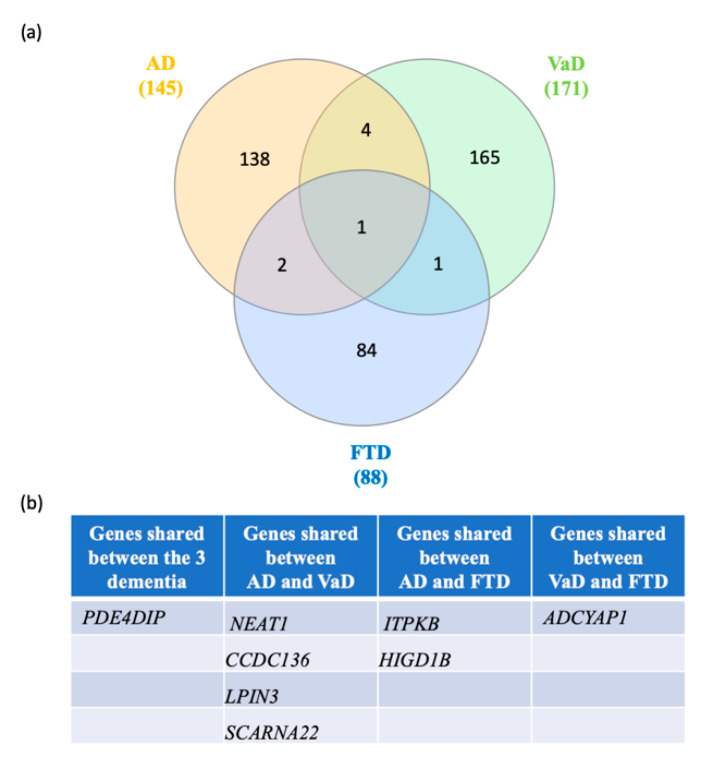
Venn diagram analysis of the switch genes shared in AD, VaD and FTD. (**a**) Venn analysis was performed using the following website: http://www.interactivenn.net/. (**b**) List of the switch genes shared between each dementia.

Next, we searched for shared switch genes between the three types of dementia. We found that phosphodiesterase 4D interacting protein (*PDE4DIP*) was the only switch gene shared among the three groups (Figure 5). Four switch genes, including small Cajal body-specific RNA 22 (*SCARNA22*), coiled-coil domain containing 136 (*CCDC136*), nuclear paraspeckle assembly transcript 1 (*NEAT1*), and lipin 3 (*LPIN3*), were shared between AD and VaD. Further, HIG1 hypoxia inducible domain family member 1B (*HIGD1B*) and type B inositol 1,4,5-trisphosphate 3-kinase (*ITPKB*) were shared between FTD and AD. Adenylate cyclase activating polypeptide 1 (*ADCYAP1*) was shared between FTD and VaD. Together, these results suggest that the clinical overlap between the dementias may be due to the expression of a few key shared switch genes.

### 2.3. Shared and Unique Biological Pathways in AD, VaD, FTD

**Figure 6:**
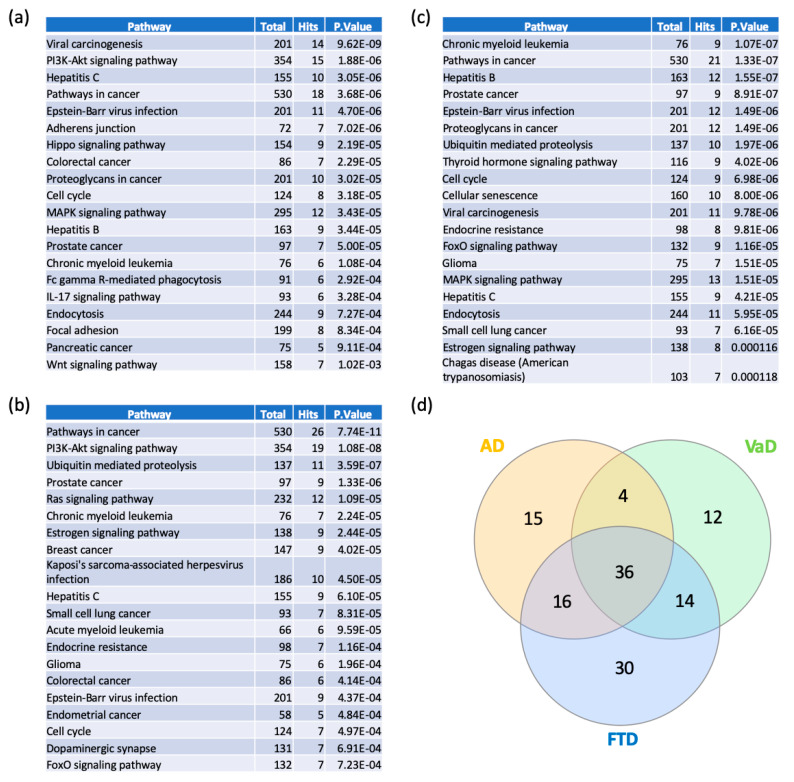
Pathway analyses of switch genes of AD, VaD and FTD patients. Pathway analysis was performed using NetworkAnalyst on tissue-specific networks derived from the protein-protein interaction database from the frontal cortex. The minimum connected network was selected for further pathway analysis. Results from the pathway analysis are derived from the Kyoto Encyclopedia of Genes and Genome (KEGG). Totals refers to the total number of genes that are known to be involved in the KEGG pathway and hits refers to the number of genes identified in this analysis that are involved in the pathway. The 20 top dysregulated pathways ranked according to the *p*-value are presented in the table. (**a**) AD pathways, (**b**) VaD pathways, (**c**) FTD pathways, and (**d**) Venn diagram analysis of the dysregulated pathways in AD, VaD and FTD.

In order to elucidate the functional and biological role of switch genes, we performed pathway analysis using NetworkAnalyst. Viral carcinogenesis and PI3K-AKT signaling pathways are the most over-represented pathways in AD (Figure 6a, Supplementary Table S5). Similarly, switch genes identified in VaD are mostly involved in cancer, PI3K-AKT, ubiquitin-mediated proteolysis (Figure 6b, Supplementary Table S5), whereas FTD is associated with cancer, Epstein–Barr virus infection, and ubiquitin-mediated proteolysis (Figure 6c, Supplementary Table S5). We found that 36 of the pathways were shared between the three dementias (Supplementary Table S6). However, several pathways were unique to each dementia (Supplementary Table S6). Indeed, 15, 12, and 30 pathways were unique to AD, VaD, and FTD, respectively. The highest ranked unique pathways were the Wnt signaling pathway, Hedgehog signaling pathway, and GnRH signaling pathway respectively for AD, VaD, and FTD.

### 2.4. Gene-Transcription Factors Interaction Analysis

**Figure 7:**
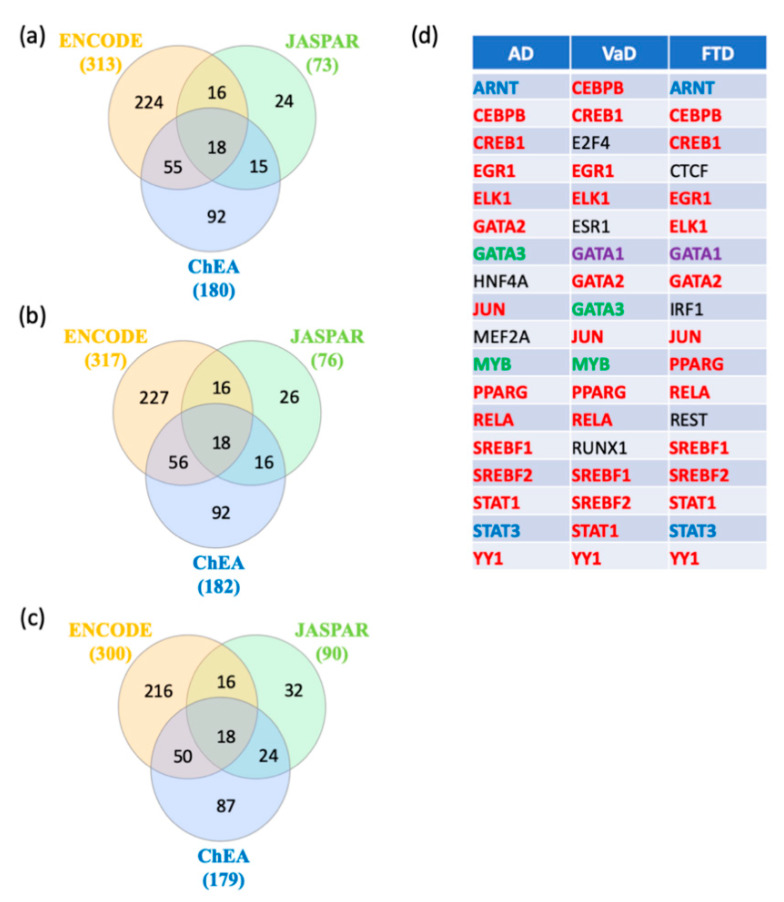
Transcription factors analysis. NetworkAnalyst was used for transcription factor analysis with ENCODE, JASPAR, and ChEA. Venn diagram analysis of the data revealed transcription factors shared by the three methods. (**a**) Transcription factors potentially regulating AD switch genes, (**b**) Transcription factors potentially regulating VaD switch genes, (**c**) Transcription factors potentially regulating FTD switch genes, and (**d**) List of the transcription factors potentially regulating AD, VaD and FTD switch genes. The transcription factors in red are shared between the 3 types of dementia. The transcription factors in green are shared between AD and VaD analysis. The transcription factors in blue are shared between AD and FTD analysis. The transcription factor in purple is shared between VaD and FTD analysis. The transcription factors in black are unique to a particular dementia.

In order to identify key regulators of switch genes obtained from AD, VaD, and FTD, gene transcription factors, interactomes were performed using NetworkAnalyst [22] with the ENCODE, JASPAR, and ChEA databases. A total of 18 shared transcription factors regulating the switch genes for each dementia were revealed by Venn analysis (Figure 7a–c). Interestingly, we observed that 12 transcription factors are shared between the three types of dementia (Figure 7d). Only two and three transcription factors were unique to VaD and FTD, respectively (Figure 7d). 

### 2.5. Protein-Chemical Interaction Analysis

**Figure 8:**
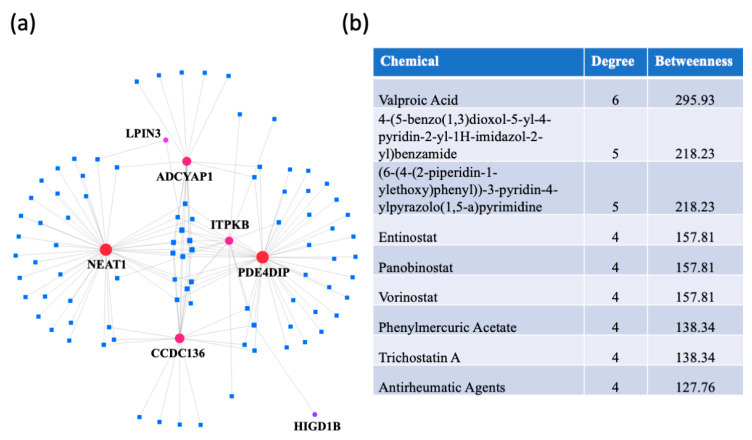
Protein-chemical interaction analysis. NetworkAnalyst was used to identify potential therapeutic reagents. (**a**) A protein-chemical network was created using the data from the Comparative Toxicogenomics Database (CTD). The dementia switch genes are represented by the red dots and the drugs are represented by blue squares. (**b**) List of the highest ranked chemicals, with a degree ≥ 4.

A protein–chemical interaction network analysis revealed drugs that are potentially useful for treating dementias. The switch genes that are shared between two or three dementias were used to create the network (Figure 8a). The highest ranked compounds, with a degree ≥ 4, are shown in Figure 8b. The most highly ranked chemical, valproic acid, was associated with NEAT1, LPIN3, ADCYAP1, CCDC136, ITPKB, and PDE4DIP. Several other chemicals were part of the HDAC inhibitor family (entinostat, panobinostat, vorinostat, trichostatine (A). In addition, antirheumatic agents were identified, suggesting a role of inflammation in dementia pathogenesis.

## 3. Discussion

### 3.1. AD, VaD and FTD Switch Genes

We investigated drastic gene expression changes associated with the transition from a healthy aging brain to that of dementia related diseases. SWIM analysis identified several switch genes associated with gene expression changes in AD, VaD, and FTD. *PDE4DIP*, also called myomegalin, is the only switch gene that is shared among the dementias. The protein encoded by PDE4DIP is responsible for anchoring PDE4D to the Golgi/centrosome of cells and promotes microtubule assembly [23,24,25]. PDE4D is a phosphohydrolase involved in signal transduction of cAMP, a key second messenger critically involved in the mechanisms underlying memory formation and cognitive processes. PDE4DIP was linked to ischemic stroke by genomic analysis [26,27,28]. PDE4DIP is a paralog of and interacts with CDK5 Regulatory Subunit Associated Protein 2 (CDK5RIP2) in vertebrates. *CDK5RAP2* expression is more abundant in the frontal cortex, the temporal cortex, and the cerebellum of AD patients [29]. Two polymorphisms regulating *CDK5RAP2* gene expression are a risk factor (rs10984186) or protective (rs4837766) for AD pathology. Pharmacological inhibition of PDE4D significantly improves memory acquisition and retrieval and prevents Aβ neurotoxic effects in mice [30]. In addition, *PDE4DIP* expression may be modified by the glucoside compound Gly-9, which inhibits the formation of prions [31]. Further investigation of PDE4D as a therapeutic target for dementia would be valuable. 

The long noncoding RNA molecule *NEAT1* was identified as a switch gene shared between AD and VaD. *NEAT1* has been linked to several neurodegenerative disorders. *NEAT1* is upregulated in Huntington’s disease and may be protective of polyglutamine repeat toxicity [32,33]. In frontotemporal dementia, *NEAT1* interacts with TDP-43, the major protein in FTD inclusion bodies [34]. More recently, *NEAT1* has been proposed as a therapeutic target for AD because knockdown of *NEAT1* is neuroprotective in an Aβ-induced cellular model [35]. Downregulation of *NEAT1* affects H3K27 acetylation (H3K27Ac) and H3K27 crotonylation (H3K27Cro) resulting in alterations in the transcriptional activity of endocytosis-related genes [36]. Collectively, these findings suggest that *NEAT1* is a possible therapeutic target for AD and the epigenetic mechanisms by which *NEAT1* may regulate Aβ clearance warrants further investigation. 

Similarly, *ITPKB* was identified a switch gene shared between AD and FTD. *ITPKB* expression increases in AD patients beyond what is observed during normal aging [37,38]. ITPKB affects calcium signaling by phosphorylating inositol 1,4,5-trisphosphate (IP3) into inositol 1,3,4,5-tetrakisphosphate (IP4). Interestingly, ITPKB colocalizes with amyloid plaques and neurofibrillary tangles in the human AD brain [39]. In addition, it has been shown that *ITPKB* overexpression may exacerbate AD pathology in an AD mouse model, whereas a catalytic inactive mutant shows no effect [40]. Interestingly, *ITPKB* expression may be regulated by the neuroprotective miR132 [39], which is downregulated in AD [41,42,43,44,45,46]. It has been proposed that the loss of miR132, and the consequent increase of ITPKB, are responsible for some AD pathology [39]. The role of ITPKB in other dementias has not yet been studied. 

Finally, *ADCYAP1* was identified as a switch gene shared between VaD and FTD. *ADCYAP1* encodes a secreted proprotein that is further processed into the neuropeptide PACAP. This pituitary neuropeptide is involved in several neurological processes such as neurodevelopment, neuroprotection, and neurogenic inflammation [47]. PACAP-deficient mice are more vulnerable to stroke [48]. During neuronal ischemic injuries, PACAP is protective via anti-apoptotic and anti-inflammatory pathways [49]. PACAP levels were significantly downregulated in several AD mouse models and in humans [50,51]. Interestingly, the decrease in PACAP was associated with clinical severity in mild cognitive impairment and AD [22]. It has been proposed that the neuroprotective role of PACAP in AD is at least partially mediated by the stimulation of α-secretase activity by PACAP [52]. Interestingly, it has been shown that treatment with PACAP decreased AD pathology by stimulating the nonamyloidogenic processing of amyloid precursor protein and increased the expression of neuroprotective factors [53,54]. More recently, intranasal administration of PACAP ameliorated the memory deficit in a Huntington’s disease model [55]. 

### 3.2. AD, VaD and FTD Switch Genes Dysregulated Pathways

Network analysis of switch genes identified viral carcinogenesis, PI3K-AKT, hepatitis C, and cancer signaling pathways as the most overrepresented pathways in all three dementias. Of note is the fact that at least 14 of the shared dysregulated pathways among the dementias are specifically cancer related and many of the other signaling pathways identified play a role in carcinogenesis. The inverse relationship between cancer and AD has been well documented for many factors and signaling pathways including PI3K-AKT [56]. Signaling pathways that are upregulated in cancer are needed to promote and sustain survival and cellular proliferation. These same pathways become downregulated in AD and other neurodegenerative diseases. 

The results presented in this study also support the fact that AD, VaD, and FTD may develop from the dysregulation of several pathways that are not shared among all three dementias. For example, the Wnt pathway was only dysregulated in AD. In the brains of healthy individuals, Wnt is involved with neuron survival, neurogenesis, synaptic plasticity, and the integrity of the blood–brain barrier [57]. Wnt signaling inhibits both Aβ production and tau phosphorylation, the hallmarks of AD pathology [57] and it block Aβ toxicity [58]. Wnt also inhibits GSK3, a kinase that phosphorylates tau [59]. In the brains of AD patients, however, Wnt signaling is reduced and linked to APOE4, a major genetic risk for the disease [60]. Lithium’s activation of Wnt signaling was shown to substantially reduce levels of Aβ in vitro and in vivo [61,62,63] and could ameliorate neurodegeneration and improve memory performance in rodents [64,65,66].

A dysregulated pathway unique for the VaD switch genes was sonic hedgehog (Shh). Our results support a recent study, which reported that DL-3-n-butylphthalide (NBP), a drug used to prevent ischemic cerebral injury and ameliorate vascular cognitive impairment, activates the Shh pathway and decreases endoplasmic reticulum stress in a rat model of VaD [67].

Dysregulation of the central regulator of cell growth and survival mTOR is another pathway unique to the VaD switch genes. The role of mTOR in VaD is well documented. In rat models of chronic cerebral hypoperfusion, the expression of mTOR and phosphorylation of the protein are reduced in pyramidal neurons and may be involved in VaD pathogenesis [68,69,70]. Interestingly, the Akt/mTOR pathway is activated in a rat model treated with Oxiracetam, a drug commonly used for the management of cerebrovascular impairments including VaD [71]. Although mTOR was not identified in our analysis as a pathway involved in AD development, several studies indicate a role for this pathway in AD etiology. Brain vascular dysfunction has been proposed as part of the mechanism that leads to several different dementias, including Lewy body dementia, Parkinson’s disease, and AD [72,73,74]. Interestingly, cerebrovascular dysfunction was detected prior to the onset of cognitive impairment in AD patients [74]. It has been proposed that rapamycin, an inhibitor of mTOR, might be useful in treating AD patients in early stages of the disease [75]. 

### 3.3. AD, VaD and FTD Switch Transcription Factors

Interestingly, most of the transcription factors identified as regulators of the dementia switch genes were shared among the three dementias, suggesting that they share several initiation mechanisms that lead to dementia. In addition, the data indicate that using therapeutic methods to target these factors would be beneficial for preventing cognitive impairment. Several of these transcription factors had been identified in previous studies to play a role in the development of dementia. PPARG, YY1, and GATA2, were identified as potential therapeutic targets for AD [76,77,78]. EGRI may play a role in the development of AD and FTD, while CREBB1, GATA2, JUN, PPARG, RELA, SREBF1 SREBF2, STAT1, and YY1 are potentially involved with the development of AD and VaD. Further, CEBPB and ELK1 are implicated in AD [12]. Comparing the results from an earlier study that focused on gene expression changes in the progression of dementia with those presented in this study, the findings strongly suggest that many of the transcription factors that may be involved in triggering dementia, particularly for AD and VaD, are also involved in the progression of the disease (Supplementary Figure S1g–i) [12]. In addition, ELK, STAT1, PPARG, YY1, CEBPB, GATA2, SREBF1, and CREB1 were identified as transcription regulators in the blood of MCI and AD and SREBF1 in AD, suggesting there is some conservation in the regulation of peripheral and central gene expression in dementia [13]. 

### 3.4. Comparison of Genes Responsible for Initiation and Progression of Dementia

In the present study, we identified genes that are potentially responsible for the switch from normal gene expression to AD, VaD and FTD. In our previous study, we analyzed gene expression changes that occur in advanced stages of each of these dementias [12]. A comparison of the results from the studies indicates that 6 AD, 78 VaD and 18 FTD genes are shared and these may play a role in both initiation and progression of the dementias (Supplementary Figure S1a–c and Table S7). Most of the genes, however, were specific to each analysis, suggesting that the unique genes play separate roles in initiation and progression in these dementias. In addition, pathway analysis indicates that 34 AD pathways, 11 VaD pathways, and 22 FTD pathways were shared between the initiation and progression of the dementias (Supplementary Figure S1d–f). Thus, although the majority of the genes that are dysregulated change from the start to the advanced stages of dementia, the pathways, in general, continue to be dysregulated throughout the course of the disease. As noted above, a comparison of the transcription factors analysis of this study to the earlier study indicates that most of the transcription factors involved in the regulation of switch genes are also involved in the regulation genes during progression of dementia (Figure S1g–i).

### 3.5. Valproic Acid May Be Useful as a Therapeutic Agent to Treat AD, VaD and FTD 

Valproic acid was identified as a potential therapeutic agent that may regulate the mutual switch genes shared among the dementias (*PDE4DIP*, *NEAT1*, *LPIN3*, *ADCYAP1*, *CCDC136*, and *ITPKB*). Valproic acid is currently prescribed to patients for seizures, bipolar disorder, and to prevent migraine headaches [79,80,81]. It has also been proposed as a potential treatment for AD because it is neuroprotective against Aβ toxicity both in vitro and in animal models [82,83]. A note of caution should be considered, however, since gender differences in learning and memory improvement were noted after valproic acid treatment in AD mice models, with males receiving a greater benefit [84]. The neuroprotective effect of valproic acid may be partially due to the inhibition of cPLA2 dependent signaling [85]. In addition, valproic acid inhibits histone deacetylase and thereby modifies gene expression [86]. In AD models, valproic acid has been shown to increase the mRNA levels of nerve growth factor and decrease the plasma level of IL1 [87]. In addition, valproic acid increases melatonin MT2 receptor expression in different hippocampal brain regions in rats [88]. Valproic acid may also be neuroprotective for Parkinson’s and prion diseases [85] and its use for the treatment of Huntington’s disease has been considered [89]. Valproic acid is currently being used as a treatment for behavioral disturbances in patients with dementia. As a monotherapy, the effect of valproic acid is limited when it is added as an adjunct therapy to drugs like quetiapine in patients who have dementia with Lewy body disease [90]. Although these results are promising, further studies are needed since a clinical study of valproic acid did not delay the agitation or psychosis observed in AD patients and magnetic resonance data indicated disease progression [91]. In addition, caution must be used in prescribing valproic acid since some patients may experience hepatic failure and patients with mitochondrial disease may be at higher risk of acute liver failure according to the drug manufacturers Lexicomp and Medline (U.S. National Library of Medicine).

### 3.6. Limitations

Because the data presented in this study are based on bioinformatic and correlational analyses, caution should be taken in interpreting these results. Ideally, the results presented here will be validated in future clinical trials with samples from patients with dementia. Unfortunately, such samples are not currently available. In addition, because this study is dependent on data collected at multiple sites, there are likely to be site-to-site differences that may influence the findings such as differences in microarray platforms, blood collection and RNA extraction methods. Other limitations may be associated with variations in diagnosis criteria. Because dementias are progressive disorders, the wide range of staging for each dementia may have impacted the results. In the studies that used samples from AD patients, the diagnostic criteria used were based solely on cognition, which may have underestimated the number of patients and potentially included healthy controls that might be preclinical for AD. Although mixed pathologies were not reported in any of the studies, disease comorbidities and medication use could have also impacted the results. 

## 4. Methods

### 4.1. Database Mining

The terms “Alzheimer’s disease”, “vascular dementia”, “frontotemporal dementia”, “brain”, “frontal cortex”, “human”, “RNA”, and “microarray” were used to search the NCBI GEO database (https://www.ncbi.nlm.nih.gov/gds) and ArrayExpress database (https://www.ebi.ac.uk/arrayexpress/) on 1 October 2019 in order to identify 18 transcriptomic data from the frontal cortex of individuals with AD, VaD, and FTD. Only studies with at least 5 samples from individuals diagnosed with dementia and controls were analyzed further. The five microarrays that met these criteria were curated using the database BaseSpace Correlation Engine (BSCE, Illumina, Inc., San Diego, CA, USA).

### 4.2. Clinical and Demographic Characteristics of Participants Included in the Study. 

The clinical and demographic information of the participants have been presented previously [12]. In each of these studies, informed consent was obtained from the study participants and study protocols were approved by the relevant ethical committees at each clinical site. The GSE122063 dataset included brain samples from 12 AD, 9 VaD, and 10 controls from the University of Michigan Alzheimer’s Disease Center Brain Bank [14]. Dementia patients and controls were matched for age, postmortem interval, and gender. The AD patients were at Braak stage III-IV and had no infarcts present in the autopsied hemisphere. VaD samples were obtained from subjects with multi-infarct dementia subtype and had no evidence or very minimal evidence of any AD typical pathology. Mixed dementia cases were removed from analysis based on pathological findings. The dataset GSE13162 was obtained from 10 sporadic FTD patients that had ubiquitinated inclusions, but no mutations in the progranulin gene (GRN), and 8 neurologically normal controls from the University of Pennsylvania Center for Neurodegenerative Disease Research Brain Bank [21]. According to the original study, age, sex, and postmortem interval to autopsy were not significantly different between cases and controls. The FTD cases were reviewed by a board-certified neuropathologist and the control brains had no evidence of neurological disease either clinically or neuropathologically [21].

### 4.3. Identification of Switch Genes by SWIM Analysis

SWItch Miner (SWIM) software, which is freely available, was used to identify switch genes using datasets from the frontal cortex of AD, VaD, and FTD subjects as previously described [11,17,18]. The switch genes identified by SWIM analysis are considered fundamental to the onset of a disease. In SWIM analysis, co-expression networks are built using the Pearson correlation coefficient between the expression of two genes. The nodes of the network are RNA transcripts and the connections between nodes represent either a significant correlation or anti-correlation of the expression of the genes. The algorithm identifies communities in the network using the k-means clustering algorithm, employing sum of squared errors (SSE) values to determine the appropriate number of clusters. It then creates a heat cartography map of the nodes according to their topological properties and extracts a set of genes, called switch genes, which are expected to mark the transition from one condition to another. In this study SWIM was used to identify genes in the frontal cortex responsible for the transition from normal cognition to dementia.

The raw datasets from the expression arrays for AD, VaD, and FTD were imported into SWIM. In the pre-processing phase, genes that are not expressed or only slightly expressed are removed. In the filtering phase, the fold-change limit was set between 1.5 and 4 in order to obtain between 1000 and 2000 genes for network analysis, and genes that were not significantly expressed differently between AD, VaD, and FTD patients compared to controls are removed. A linear fold change of 2 was used as the cut-off value for the GSE122063 and 1.5-fold change for GSE13162 and GSE118553. A false discovery rate method was applied to correct for multiple tests and a Pearson correlation analysis was used to build a co-expression network of genes differentially expressed between AD, VaD, and FTD patients and controls as previously described [11]. The k-means algorithm was then used to identify communities within the network [11]. To determine the number of clusters, SWIM uses Scree plot, which allows replicating the clustering many times with a new set of initial cluster centroid positions, and for each replicate the k-means algorithm performs iterations until the minimum of the SSE function is reached. The cluster configuration with the lowest SSE values among the replicates is designated as the number of clusters. The heat cartography map is built using a clusterphobic coefficient *Kπ*, which measures external and internal node connections, and the global within-module degree *Zg*, which measures the extent each node is connected to others in its own community. A node is considered a hub when *Zg* exceeds 5. The average Pearson correlation coefficient (APCC) between the expression profiles of each node and its nearest neighbors is used to build the heat cartography map. Using APCC, three types of hubs may be identified. Date hubs show low positive co-expression with their partners (low APCC), party hubs show high positive co-expression (high APCC), and nodes that have negative APCC values are called fight club hubs [18]. In the final step of SWIM analysis, switch genes are identified that are a subset of the fight club hubs that interact outside of their community. Switch genes are characterized as not being a hub in their own cluster (low *Zg* < 2.5), having many links outside their own cluster (*Kπ* > 0.8, when *Kπ* is close to 1 most of its links are external to its own module), and having a negative average weight of incident links (APCC < 0) [18].

### 4.4. Network and Pathway Analysis 

Entrez gene identifiers for the switch genes were imported into NetworkAnalyst, https://www.networkanalyst.ca/ [92]. For network analysis, the tissue-specific data from DifferentialNet (http://netbio.bgu.ac.il/diffnet/ [93] derived from the protein–protein interaction database from frontal cortex was used. The minimum connected network was selected for further pathway analysis. For pathway analysis, data derived from the Kyoto Encyclopedia of Genes and Genome (KEGG) was used.

### 4.5. Transcription Factor Analysis 

Network analysis of transcription factors that potentially regulate the switch genes was done using NetworkAnalyst. Transcription factor and gene target data were derived from the Encyclopedia of DNA Elements (ENCODE) ChIP-seq data, ChIP Enrichment Analysis (ChEA), or JASPAR database [94,95,96]. ENCODE uses the BETA Minus Algorithm in which only peak intensity signal <500 and the predicted regulatory potential score <1 is used. ChEA transcription factor targets database inferred from integrating literature curated Chip-X data. JASPAR is an open-access database of curated, non-redundant transcription factor (TF)-binding profiles. Transcription factors were ranked according to network topology measurements, including degree and betweenness centrality. A Venn diagram analysis was performed with the transcription factors identified with each database. 

### 4.6. Genes-Chemical Interaction

Switch genes that are shared among the dementias were imported into NetworkAnalyst for protein–chemical interaction analysis. Data from the Comparative Toxicogenomics database (CTD) downloaded in November 2016 was used for the analysis [97,98,99,100].

## 5. Conclusions

In this study, the disruption of the regulation of PDE4DIP expression was identified as a key early event that may lead to the development of AD, VaD, and FTD. The major pathways that are dysregulated in these dementias are inflammation, PI3K-AKT, and ubiquitin-mediated proteolysis. The data show that a group of 12 transcription factors regulate gene expression in all three dementias. In addition, unique dysregulated gene expression, pathways, and transcription factors were identified for each of the dementias. Valproic acid was identified as a possible neuroprotective therapeutic useful for delaying the onset of AD, VaD, and FTD. 

## Figures and Tables

**Table 1:** Gene expression datasets selected in this study.

Dataset	Phenotype	Brain Region	Cases/Controls	Platform	PMID
GSE122063	Alzheimer’s disease	Frontal cortex	12/11	Agilent Human 8x60k v2 microarrays	[14]
GSE118553	Alzheimer’s disease	Frontal cortex	52/27	Illumina HumanHT-12 V4.0 expression beadchip	[19]
GSE84422	Alzheimer’s disease	Frontal cortex	21/11	Affymetrix GeneChip Human HG_U133 Plus 2.0	[20]
GSE122063	Vascular dementia	Frontal cortex	8/11	Agilent Human 8x60k v2 microarrays	[14]
GSE13162	Frontotemporal dementia	Frontal cortex	10/8	Affymetrix GeneChip Human HG_U133A version	[21]

## Supplementary Materials

The following are available online at https://www.mdpi.com/1422-0067/21/11/3787/s1, Table S1. Switch genes identified in the frontal cortex of AD subjects compared to non-demented controls (GSE122063). Table S2. Switch genes identified in the frontal cortex of VaD subjects compared to non-demented controls (GSE122063). Table S3. Switch genes identified in the frontal cortex of FTD subjects compared to non-demented controls (GSE13162). Table S4. Shared and unique switch genes in the dementia. Table S5. Pathway analysis of switch genes in the frontal cortex of AD, VaD and FTD subjects. Table S6. Shared and unique pathways. Table S7. Comparison of the genes, pathways and transcription factors during the initiation and progression of dementia. Figure S1. Venn Diagram analysis of the genes, pathways and transcription factors during the initiation and progression of dementia. (a–c) Venn diagram analysis of differentially expressed genes (DEG) and Switch genes in AD (a), VaD (b) and FTD (c). (d–f) Venn diagram analysis of pathways identified from differentially expressed genes (DEG) and Switch genes in AD (d), VaD (e) and FTD (f). (g–i) Venn diagram analysis of transcription factors identified from differentially expressed genes (DEG) and Switch genes in AD (g), VaD (h) and FTD (i).

## References

[1] WHO (2019). In Risk Reduction of Cognitive Decline and Dementia: WHO Guidelines.

[2] Habes M., Grothe M.J., Tunc B., McMillan C., Wolk D.A., Davatzikos C. (2020). Disentangling Heterogeneity in Alzheimer’s Disease and Related Dementias Using Data-Driven Methods. Biol. Psychiatry.

[3] Matej R., Tesar A., Rusina R. (2019). Alzheimer’s disease and other neurodegenerative dementias in comorbidity: A clinical and neuropathological overview. Clin. Biochem..

[4] Gallardo G., Holtzman D.M. (2019). Amyloid-beta and Tau at the Crossroads of Alzheimer’s Disease. Adv. Exp. Med. Biol..

[5] Blennow K., Zetterberg H. (2018). Biomarkers for Alzheimer’s disease: Current status and prospects for the future. J. Intern. Med..

[6] O’Brien J.T., Thomas A. (2015). Vascular dementia. Lancet.

[7] Toledo J.B., Arnold S.E., Raible K., Brettschneider J., Xie S.X., Grossman M., Monsell S.E., Kukull W.A., Trojanowski J.Q. (2013). Contribution of cerebrovascular disease in autopsy confirmed neurodegenerative disease cases in the National Alzheimer’s Coordinating Centre. Brain.

[8] Johnen A., Bertoux M. (2019). Psychological and Cognitive Markers of Behavioral Variant Frontotemporal Dementia-A Clinical Neuropsychologist’s View on Diagnostic Criteria and Beyond. Front. Neurol..

[9] Harciarek M., Jodzio K. (2005). Neuropsychological differences between frontotemporal dementia and Alzheimer’s disease: A review. Neuropsychol. Rev..

[10] Lanke V., Moolamalla S.T.R., Roy D., Vinod P.K. (2018). Integrative Analysis of Hippocampus Gene Expression Profiles Identifies Network Alterations in Aging and Alzheimer’s Disease. Front. Aging Neurosci..

[11] Potashkin J.A., Bottero V., Santiago J.A., Quinn J.P. (2019). Computational identification of key genes that may regulate gene expression reprogramming in Alzheimer’s patients. PLoS ONE.

[12] Santiago J.A., Bottero V., Potashkin J.A. (2020). Transcriptomic and Network Analysis Identifies Shared and Unique Pathways across Dementia Spectrum Disorders. Int. J. Mol. Sci..

[13] Bottero V., Potashkin J.A. (2019). Meta-Analysis of Gene Expression Changes in the Blood of Patients with Mild Cognitive Impairment and Alzheimer’s Disease Dementia. Int. J. Mol. Sci..

[14] McKay E.C., Beck J.S., Khoo S.K., Dykema K.J., Cottingham S.L., Winn M.E., Paulson H.L., Lieberman A.P., Counts S.E. (2019). Peri-Infarct Upregulation of the Oxytocin Receptor in Vascular Dementia. J. Neuropathol. Exp. Neurol..

[15] Ferrari R., Forabosco P., Vandrovcova J., Botia J.A., Guelfi S., Warren J.D., Consortium U.K.B.E., Momeni P., Weale M.E., Ryten M. (2016). Frontotemporal dementia: Insights into the biological underpinnings of disease through gene co-expression network analysis. Mol. Neurodegener..

[16] Ferrari R., Lovering R.C., Hardy J., Lewis P.A., Manzoni C. (2017). Weighted Protein Interaction Network Analysis of Frontotemporal Dementia. J. Proteome Res..

[17] Paci P., Colombo T., Fiscon G., Gurtner A., Pavesi G., Farina L. (2017). SWIM: A computational tool to unveiling crucial nodes in complex biological networks. Sci. Rep..

[18] Fiscon G., Conte F., Farina L., Paci P. (2018). Network-Based Approaches to Explore Complex Biological Systems towards Network Medicine. Genes.

[19] Patel H., Hodges A.K., Curtis C., Lee S.H., Troakes C., Dobson R.J.B., Newhouse S.J. (2019). Transcriptomic analysis of probable asymptomatic and symptomatic alzheimer brains. Brain Behav. Immun..

[20] Wang M., Roussos P., McKenzie A., Zhou X., Kajiwara Y., Brennand K.J., De Luca G.C., Crary J.F., Casaccia P., Buxbaum J.D. (2016). Integrative network analysis of nineteen brain regions identifies molecular signatures and networks underlying selective regional vulnerability to Alzheimer’s disease. Genome Med..

[21] Chen-Plotkin A.S., Geser F., Plotkin J.B., Clark C.M., Kwong L.K., Yuan W., Grossman M., Van Deerlin V.M., Trojanowski J.Q., Lee V.M. (2008). Variations in the progranulin gene affect global gene expression in frontotemporal lobar degeneration. Hum. Mol. Genet..

[22] Han P., Caselli R.J., Baxter L., Serrano G., Yin J., Beach T.G., Reiman E.M., Shi J. (2015). Association of pituitary adenylate cyclase-activating polypeptide with cognitive decline in mild cognitive impairment due to Alzheimer disease. JAMA Neurol..

[23] Wang Z., Zhang C., Qi R.Z. (2014). A newly identified myomegalin isoform functions in Golgi microtubule organization and ER-Golgi transport. J. Cell Sci..

[24] Verde I., Pahlke G., Salanova M., Zhang G., Wang S., Coletti D., Onuffer J., Jin S.L., Conti M. (2001). Myomegalin is a novel protein of the golgi/centrosome that interacts with a cyclic nucleotide phosphodiesterase. J. Biol. Chem..

[25] Roubin R., Acquaviva C., Chevrier V., Sedjai F., Zyss D., Birnbaum D., Rosnet O. (2013). Myomegalin is necessary for the formation of centrosomal and Golgi-derived microtubules. Biol. Open.

[26] Gretarsdottir S., Thorleifsson G., Reynisdottir S.T., Manolescu A., Jonsdottir S., Jonsdottir T., Gudmundsdottir T., Bjarnadottir S.M., Einarsson O.B., Gudjonsdottir H.M. (2003). The gene encoding phosphodiesterase 4D confers risk of ischemic stroke. Nat. Genet..

[27] Yoon D., Park S.K., Kang D., Park T., Park J.W. (2011). Meta-analysis of homogeneous subgroups reveals association between PDE4D gene variants and ischemic stroke. Neuroepidemiology.

[28] Auer P.L., Nalls M., Meschia J.F., Worrall B.B., Longstreth W.T., Seshadri S., Kooperberg C., Burger K.M., Carlson C.S., Carty C.L. (2015). Rare and Coding Region Genetic Variants Associated With Risk of Ischemic Stroke: The NHLBI Exome Sequence Project. JAMA Neurol..

[29] Miron J., Picard C., Nilsson N., Frappier J., Dea D., Theroux L., Poirier J., Alzheimer’s Disease Neuroimaging Initiative, United Kingdom Brain Expression Consortium (2018). CDK5RAP2 gene and tau pathophysiology in late-onset sporadic Alzheimer’s disease. Alzheimer’ Dement..

[30] Cui S.Y., Yang M.X., Zhang Y.H., Zheng V., Zhang H.T., Gurney M.E., Xu Y., O’Donnell J.M. (2019). Protection from Amyloid beta Peptide-Induced Memory, Biochemical, and Morphological Deficits by a Phosphodiesterase-4D Allosteric Inhibitor. J. Pharmacol. Exp. Ther..

[31] Nishizawa K., Oguma A., Kawata M., Sakasegawa Y., Teruya K., Doh-ura K. (2014). Efficacy and mechanism of a glycoside compound inhibiting abnormal prion protein formation in prion-infected cells: Implications of interferon and phosphodiesterase 4D-interacting protein. J. Virol..

[32] Sunwoo J.S., Lee S.T., Im W., Lee M., Byun J.I., Jung K.H., Park K.I., Jung K.Y., Lee S.K., Chu K. (2017). Altered Expression of the Long Noncoding RNA NEAT1 in Huntington’s Disease. Mol. Neurobiol..

[33] Cheng C., Spengler R.M., Keiser M.S., Monteys A.M., Rieders J.M., Ramachandran S., Davidson B.L. (2018). The long non-coding RNA NEAT1 is elevated in polyglutamine repeat expansion diseases and protects from disease gene-dependent toxicities. Hum. Mol. Genet..

[34] Tollervey J.R., Curk T., Rogelj B., Briese M., Cereda M., Kayikci M., Konig J., Hortobagyi T., Nishimura A.L., Zupunski V. (2011). Characterizing the RNA targets and position-dependent splicing regulation by TDP-43. Nat. Neurosci..

[35] Zhao M.Y., Wang G.Q., Wang N.N., Yu Q.Y., Liu R.L., Shi W.Q. (2019). The long-non-coding RNA NEAT1 is a novel target for Alzheimer’s disease progression via miR-124/BACE1 axis. Neurol. Res..

[36] Wang Z., Zhao Y., Xu N., Zhang S., Wang S., Mao Y., Zhu Y., Li B., Jiang Y., Tan Y. (2019). NEAT1 regulates neuroglial cell mediating Abeta clearance via the epigenetic regulation of endocytosis-related genes expression. Cell. Mol. Life Sci..

[37] Saetre P., Jazin E., Emilsson L. (2011). Age-related changes in gene expression are accelerated in Alzheimer’s disease. Synapse.

[38] Emilsson L., Saetre P., Jazin E. (2006). Alzheimer’s disease: mRNA expression profiles of multiple patients show alterations of genes involved with calcium signaling. Neurobiol. Dis..

[39] Salta E., Sierksma A., Vanden Eynden E., De Strooper B. (2016). miR-132 loss de-represses ITPKB and aggravates amyloid and TAU pathology in Alzheimer’s brain. EMBO Mol. Med..

[40] Stygelbout V., Leroy K., Pouillon V., Ando K., D’Amico E., Jia Y., Luo H.R., Duyckaerts C., Erneux C., Schurmans S. (2014). Inositol trisphosphate 3-kinase B is increased in human Alzheimer brain and exacerbates mouse Alzheimer pathology. Brain.

[41] Cogswell J.P., Ward J., Taylor I.A., Waters M., Shi Y., Cannon B., Kelnar K., Kemppainen J., Brown D., Chen C. (2008). Identification of miRNA changes in Alzheimer’s disease brain and CSF yields putative biomarkers and insights into disease pathways. J. Alzheimer’ Dis..

[42] Dorval V., Nelson P.T., Hebert S.S. (2013). Circulating microRNAs in Alzheimer’s disease: The search for novel biomarkers. Front. Mol. Neurosci..

[43] Hebert S.S., Wang W.X., Zhu Q., Nelson P.T. (2013). A study of small RNAs from cerebral neocortex of pathology-verified Alzheimer’s disease, dementia with lewy bodies, hippocampal sclerosis, frontotemporal lobar dementia, and non-demented human controls. J. Alzheimer’ Dis..

[44] Lau P., Bossers K., Janky R., Salta E., Frigerio C.S., Barbash S., Rothman R., Sierksma A.S., Thathiah A., Greenberg D. (2013). Alteration of the microRNA network during the progression of Alzheimer’s disease. EMBO Mol. Med..

[45] Wong H.K., Veremeyko T., Patel N., Lemere C.A., Walsh D.M., Esau C., Vanderburg C., Krichevsky A.M. (2013). De-repression of FOXO3a death axis by microRNA-132 and -212 causes neuronal apoptosis in Alzheimer’s disease. Hum. Mol. Genet..

[46] Smith P.Y., Hernandez-Rapp J., Jolivette F., Lecours C., Bisht K., Goupil C., Dorval V., Parsi S., Morin F., Planel E. (2015). miR-132/212 deficiency impairs tau metabolism and promotes pathological aggregation in vivo. Hum. Mol. Genet..

[47] Vaudry D., Gonzalez B.J., Basille M., Yon L., Fournier A., Vaudry H. (2000). Pituitary adenylate cyclase-activating polypeptide and its receptors: From structure to functions. Pharmacol. Rev..

[48] Chen Y., Samal B., Hamelink C.R., Xiang C.C., Chen Y., Chen M., Vaudry D., Brownstein M.J., Hallenbeck J.M., Eiden L.E. (2006). Neuroprotection by endogenous and exogenous PACAP following stroke. Regul. Pept..

[49] Reglodi D., Vaczy A., Rubio-Beltran E., MaassenVanDenBrink A. (2018). Protective effects of PACAP in ischemia. J. Headache Pain.

[50] Wu Z.L., Ciallella J.R., Flood D.G., O’Kane T.M., Bozyczko-Coyne D., Savage M.J. (2006). Comparative analysis of cortical gene expression in mouse models of Alzheimer’s disease. Neurobiol. Aging.

[51] Han P., Liang W., Baxter L.C., Yin J., Tang Z., Beach T.G., Caselli R.J., Reiman E.M., Shi J. (2014). Pituitary adenylate cyclase-activating polypeptide is reduced in Alzheimer disease. Neurology.

[52] Kojro E., Postina R., Buro C., Meiringer C., Gehrig-Burger K., Fahrenholz F. (2006). The neuropeptide PACAP promotes the alpha-secretase pathway for processing the Alzheimer amyloid precursor protein. FASEB J..

[53] Han P., Tang Z., Yin J., Maalouf M., Beach T.G., Reiman E.M., Shi J. (2014). Pituitary adenylate cyclase-activating polypeptide protects against beta-amyloid toxicity. Neurobiol. Aging.

[54] Rat D., Schmitt U., Tippmann F., Dewachter I., Theunis C., Wieczerzak E., Postina R., Van Leuven F., Fahrenholz F., Kojro E. (2011). Neuropeptide pituitary adenylate cyclase-activating polypeptide (PACAP) slows down Alzheimer’s disease-like pathology in amyloid precursor protein-transgenic mice. FASEB J..

[55] Cabezas-Llobet N., Vidal-Sancho L., Masana M., Fournier A., Alberch J., Vaudry D., Xifro X. (2018). Pituitary Adenylate Cyclase-Activating Polypeptide (PACAP) Enhances Hippocampal Synaptic Plasticity and Improves Memory Performance in Huntington’s Disease. Mol. Neurobiol..

[56] Shafi O. (2016). Inverse relationship between Alzheimer’s disease and cancer, and other factors contributing to Alzheimer’s disease: A systematic review. BMC Neurol..

[57] Jia L., Pina-Crespo J., Li Y. (2019). Restoring Wnt/beta-catenin signaling is a promising therapeutic strategy for Alzheimer’s disease. Mol. Brain.

[58] Cerpa W., Farias G.G., Godoy J.A., Fuenzalida M., Bonansco C., Inestrosa N.C. (2010). Wnt-5a occludes Abeta oligomer-induced depression of glutamatergic transmission in hippocampal neurons. Mol. Neurodegener..

[59] Qiu S., Korwek K.M., Weeber E.J. (2006). A fresh look at an ancient receptor family: Emerging roles for low density lipoprotein receptors in synaptic plasticity and memory formation. Neurobiol. Learn. Mem..

[60] Caruso A., Motolese M., Iacovelli L., Caraci F., Copani A., Nicoletti F., Terstappen G.C., Gaviraghi G., Caricasole A. (2006). Inhibition of the canonical Wnt signaling pathway by apolipoprotein E4 in PC12 cells. J. Neurochem..

[61] Phiel C.J., Wilson C.A., Lee V.M., Klein P.S. (2003). GSK-3alpha regulates production of Alzheimer’s disease amyloid-beta peptides. Nature.

[62] Su Y., Ryder J., Li B., Wu X., Fox N., Solenberg P., Brune K., Paul S., Zhou Y., Liu F. (2004). Lithium, a common drug for bipolar disorder treatment, regulates amyloid-beta precursor protein processing. Biochemistry.

[63] Toledo E.M., Inestrosa N.C. (2010). Activation of Wnt signaling by lithium and rosiglitazone reduced spatial memory impairment and neurodegeneration in brains of an APPswe/PSEN1DeltaE9 mouse model of Alzheimer’s disease. Mol. Psychiatry.

[64] Fiorentini A., Rosi M.C., Grossi C., Luccarini I., Casamenti F. (2010). Lithium improves hippocampal neurogenesis, neuropathology and cognitive functions in APP mutant mice. PLoS ONE.

[65] De Ferrari G.V., Chacon M.A., Barria M.I., Garrido J.L., Godoy J.A., Olivares G., Reyes A.E., Alvarez A., Bronfman M., Inestrosa N.C. (2003). Activation of Wnt signaling rescues neurodegeneration and behavioral impairments induced by beta-amyloid fibrils. Mol. Psychiatry.

[66] Caccamo A., Oddo S., Tran L.X., LaFerla F.M. (2007). Lithium reduces tau phosphorylation but not A beta or working memory deficits in a transgenic model with both plaques and tangles. Am. J. Pathol..

[67] Niu X.L., Jiang X., Xu G.D., Zheng G.M., Tang Z.P., Yin N., Li X.Q., Yang Y.Y., Lv P.Y. (2019). DL-3-n-butylphthalide alleviates vascular cognitive impairment by regulating endoplasmic reticulum stress and the Shh/Ptch1 signaling-pathway in rats. J. Cell. Physiol..

[68] Zhu Y., Zeng Y., Wang X., Ye X. (2013). Effect of electroacupuncture on the expression of mTOR and eIF4E in hippocampus of rats with vascular dementia. Neurol. Sci..

[69] Jia Y., Jin W., Xiao Y., Dong Y., Wang T., Fan M., Xu J., Meng N., Li L., Lv P. (2015). Lipoxin A4 methyl ester alleviates vascular cognition impairment by regulating the expression of proteins related to autophagy and ER stress in the rat hippocampus. Cell. Mol. Biol. Lett..

[70] Park J.A., Lee C.H. (2017). Temporal changes in mammalian target of rapamycin (mTOR) and phosphorylated-mTOR expressions in the hippocampal CA1 region of rat with vascular dementia. J. Vet. Sci..

[71] Xu J., Qi Q., Lv P., Dong Y., Jiang X., Liu Z. (2019). Oxiracetam ameliorates cognitive deficits in vascular dementia rats by regulating the expression of neuronal apoptosis/autophagy-related genes associated with the activation of the Akt/mTOR signaling pathway. Braz. J. Med. Biol. Res..

[72] Binnewijzend M.A., Kuijer J.P., Van der Flier W.M., Benedictus M.R., Moller C.M., Pijnenburg Y.A., Lemstra A.W., Prins N.D., Wattjes M.P., Van Berckel B.N. (2014). Distinct perfusion patterns in Alzheimer’s disease, frontotemporal dementia and dementia with Lewy bodies. Eur. Radiol..

[73] Syrimi Z.J., Vojtisek L., Eliasova I., Viskova J., Svatkova A., Vanicek J., Rektorova I. (2017). Arterial spin labelling detects posterior cortical hypoperfusion in non-demented patients with Parkinson’s disease. J. Neural. Transm..

[74] Iturria-Medina Y., Sotero R.C., Toussaint P.J., Mateos-Perez J.M., Evans A.C., Alzheimer’s Disease Neuroimaging I. (2016). Early role of vascular dysregulation on late-onset Alzheimer’s disease based on multifactorial data-driven analysis. Nat. Commun..

[75] Carosi J.M., Sargeant T.J. (2019). Rapamycin and Alzheimer disease: A double-edged sword?. Autophagy.

[76] Nowak K., Lange-Dohna C., Zeitschel U., Gunther A., Luscher B., Robitzki A., Perez-Polo R., Rossner S. (2006). The transcription factor Yin Yang 1 is an activator of BACE1 expression. J. Neurochem..

[77] Rahman M.R., Islam T., Turanli B., Zaman T., Faruquee H.M., Rahman M.M., Mollah M.N.H., Nanda R.K., Arga K.Y., Gov E. (2019). Network-based approach to identify molecular signatures and therapeutic agents in Alzheimer’s disease. Comput. Biol. Chem..

[78] Rahman M.R., Islam T., Zaman T., Shahjaman M., Karim M.R., Huq F., Quinn J.M.W., Holsinger R.M.D., Gov E., Moni M.A. (2020). Identification of molecular signatures and pathways to identify novel therapeutic targets in Alzheimer’s disease: Insights from a systems biomedicine perspective. Genomics.

[79] Tariot P.N., Loy R., Ryan J.M., Porsteinsson A., Ismail S. (2002). Mood stabilizers in Alzheimer’s disease: Symptomatic and neuroprotective rationales. Adv. Drug Deliv. Rev..

[80] Loy R., Tariot P.N. (2002). Neuroprotective properties of valproate: Potential benefit for AD and tauopathies. J. Mol. Neurosci..

[81] Zhang X.Z., Li X.J., Zhang H.Y. (2010). Valproic acid as a promising agent to combat Alzheimer’s disease. Brain Res. Bull..

[82] Long Z., Zheng M., Zhao L., Xie P., Song C., Chu Y., Song W., He G. (2013). Valproic acid attenuates neuronal loss in the brain of APP/PS1 double transgenic Alzheimer’s disease mice model. Curr. Alzheimer Res..

[83] Zhao L., Zhu L., Guo X. (2018). Valproic acid attenuates Abeta25-35-induced neurotoxicity in PC12 cells through suppression of mitochondria-mediated apoptotic pathway. Biomed. Pharmacother..

[84] Long Z., Zeng Q., Wang K., Sharma A., He G. (2016). Gender difference in valproic acid-induced neuroprotective effects on APP/PS1 double transgenic mice modeling Alzheimer’s disease. Acta Biochim. Biophys. Sin..

[85] Williams R.S., Bate C. (2016). An in vitro model for synaptic loss in neurodegenerative diseases suggests a neuroprotective role for valproic acid via inhibition of cPLA2 dependent signalling. Neuropharmacology.

[86] Gottlicher M., Minucci S., Zhu P., Kramer O.H., Schimpf A., Giavara S., Sleeman J.P., Lo Coco F., Nervi C., Pelicci P.G. (2001). Valproic acid defines a novel class of HDAC inhibitors inducing differentiation of transformed cells. EMBO J..

[87] Noh H., Seo H. (2014). Age-dependent effects of valproic acid in Alzheimer’s disease (AD) mice are associated with nerve growth factor (NGF) regulation. Neuroscience.

[88] Bahna S.G., Sathiyapalan A., Foster J.A., Niles L.P. (2014). Regional upregulation of hippocampal melatonin MT2 receptors by valproic acid: Therapeutic implications for Alzheimer’s disease. Neurosci. Lett..

[89] Scheuing L., Chiu C.T., Liao H.M., Linares G.R., Chuang D.M. (2014). Preclinical and clinical investigations of mood stabilizers for Huntington’s disease: What have we learned?. Int. J. Biol. Sci..

[90] Dolder C.R., Nealy K.L., McKinsey J. (2012). Valproic acid in dementia: Does an optimal dose exist?. J. Pharm. Pract..

[91] Lauterbach E.C., Victoroff J., Coburn K.L., Shillcutt S.D., Doonan S.M., Mendez M.F. (2010). Psychopharmacological neuroprotection in neurodegenerative disease: Assessing the preclinical data. J. Neuropsychiatry Clin. Neurosci..

[92] Xia J., Gill E.E., Hancock R.E. (2015). NetworkAnalyst for statistical, visual and network-based meta-analysis of gene expression data. Nat. Protoc..

[93] Basha O., Shpringer R., Argov C.M., Yeger-Lotem E. (2018). The DifferentialNet database of differential protein-protein interactions in human tissues. Nucleic Acids Res..

[94] Consortium E.P. (2011). A user’s guide to the encyclopedia of DNA elements (ENCODE). PLoS Biol..

[95] Lachmann A., Xu H., Krishnan J., Berger S.I., Mazloom A.R., Ma’ayan A. (2010). ChEA: Transcription factor regulation inferred from integrating genome-wide ChIP-X experiments. Bioinformatics.

[96] Khan A., Fornes O., Stigliani A., Gheorghe M., Castro-Mondragon J.A., Van der Lee R., Bessy A., Cheneby J., Kulkarni S.R., Tan G. (2018). JASPAR 2018: Update of the open-access database of transcription factor binding profiles and its web framework. Nucleic Acids Res..

[97] Mattingly C.J., Colby G.T., Forrest J.N., Boyer J.L. (2003). The Comparative Toxicogenomics Database (CTD). Environ. Health Perspect..

[98] Davis A.P., King B.L., Mockus S., Murphy C.G., Saraceni-Richards C., Rosenstein M., Wiegers T., Mattingly C.J. (2011). The Comparative Toxicogenomics Database: Update 2011. Nucleic Acids Res..

[99] Davis A.P., Murphy C.G., Johnson R., Lay J.M., Lennon-Hopkins K., Saraceni-Richards C., Sciaky D., King B.L., Rosenstein M.C., Wiegers T.C. (2013). The Comparative Toxicogenomics Database: Update 2013. Nucleic Acids Res..

[100] Davis A.P., Grondin C.J., Lennon-Hopkins K., Saraceni-Richards C., Sciaky D., King B.L., Wiegers T.C., Mattingly C.J. (2015). The Comparative Toxicogenomics Database’s 10th year anniversary: Update 2015. Nucleic Acids Res..

